# Role of Peritoneal Macrophages in Cytomegalovirus-induced Acceleration of Autoimmune Diabetes in BB-rats

**DOI:** 10.1080/10446670310001626517

**Published:** 2003

**Authors:** Jan-Luuk Hillebrands, Nienke van der Werf, Flip A. Klatter, Cathrien A. Bruggeman, Jan Rozing

**Affiliations:** 1 Faculty of Medical Sciences Immunology Section, Department of Cell Biology University of Groningen A. Deusinglaan 1 Groningen NL-9713 AV The Netherlands; 2 Department of Medical Microbiology University Hospital Maastricht P. Deleyelaan 25 Maastricht NL-6202 AZ The Netherlands

## Abstract

*Background*: As one of the natural perturbants, infection with cytomegalovirus (CMV) is believed to play a role in the development of Type I diabetes. Using the DP-BB rat model for autoimmune diabetes, we here report about possible mechanisms responsible for R(at)CMV-induced accelerated onset of diabetes.

*Methods*: Rats were i.p. infected with 2 × 10^6^ plaque forming units (pfu) RCMV and followed for diabetes development. Presence of RCMV antigens and DNA was analyzed by immunohistochemistry and PCR on pancreatic tissue and isolated islets. The effect of viral infection on peritoneal macrophages (pMΦ) and diabetes development was studied by analyzing numbers of pMΦ, virus permissiveness and by depletion of this subset by peritoneal lavage.

*Results*: RCMV accelerated onset of diabetes without infecting pancreatic islets. Immunohistochemistry and PCR on pancreas and isolated islets indicated that islets are non-permissive for RCMV. Infection results in an influx of pMΦ 1 day p.i. of which ~0.05% showed signs of reproductive infection. Depletion of pMΦ on days 1-3 p.i. completely counteracted the accelerating effect of RCMV.

*Interpretation*: RCMV accelerates onset of diabetes without infecting pancreatic islets. pMΦ might function as an carriage to disseminate virus to the pancreas where they enhance activation of autoreactive T cells resulting in accelerated onset of diabetes.


*Clinical & Developmental Immunology*, June–December 2003 VOL. 10 (2–4), pp. 133–139

Taylor & Francis healthsciences

### Role of Peritoneal Macrophages in Cytomegalovirus-induced Acceleration of Autoimmune Diabetes in BB-rats

JAN-LUUK HILLEBRANDS[a,*], NIENKE VAN DER WERF[a,*], FLIP A. KLATTER[a], CATHRIEN A. BRUGGEMAN[b] and JAN ROZING[a,†]

[a]*Faculty of Medical Sciences, Immunology Section, Department of Cell Biology, University of Groningen, A. Deusinglaan 1, NL-9713 AV, Groningen, The Netherlands;* [b]*Department of Medical Microbiology, University Hospital Maastricht, P. Deleyelaan 25, NL-6202 AZ, Maastricht, The Netherlands*

*Background*: As one of the natural perturbants, infection with cytomegalovirus (CMV) is believed to play a role in the development of Type I diabetes. Using the DP-BB rat model for autoimmune diabetes, we here report about possible mechanisms responsible for R(at)CMV-induced accelerated onset of diabetes.

*Methods*: Rats were i.p. infected with $2 \times 10^6$ plaque forming units (pfu) RCMV and followed for diabetes development. Presence of RCMV antigens and DNA was analyzed by immunohistochemistry and PCR on pancreatic tissue and isolated islets. The effect of viral infection on peritoneal macrophages (pMΦ) and diabetes development was studied by analyzing numbers of pMΦ, virus permissiveness and by depletion of this subset by peritoneal lavage.

*Results*: RCMV accelerated onset of diabetes without infecting pancreatic islets. Immunohistochemistry and PCR on pancreas and isolated islets indicated that islets are non-permissive for RCMV. Infection results in an influx of pMΦ 1 day p.i. of which ~0.05% showed signs of reproductive infection. Depletion of pMΦ on days 1–3 p.i. completely counteracted the accelerating effect of RCMV.

*Interpretation*: RCMV accelerates onset of diabetes without infecting pancreatic islets. pMΦ might function as an carriage to disseminate virus to the pancreas where they enhance activation of autoreactive T cells resulting in accelerated onset of diabetes.

*Keywords*: BB rats; Cytomegalovirus; Diabetes; Macrophages; RCMV; Virus

### INTRODUCTION

Type 1 diabetes or Insulin Dependent Diabetes Mellitus (IDDM) (accounting for 10% of all cases of diabetes) is an organ-specific autoimmune disease, which results from the selective destruction of pancreatic β-cells. This destruction is histologically characterized by an inflammatory infiltrate around or within the islets (insulitis) containing large numbers of mononuclear cells and $CD8^+$ T cells (Notkins and Lernmark, 2001). Clinically, Type 1 diabetes presents as rapid onset polyuria, polydipsia, polyphagia, weight loss and hyperglycaemia (Greiner *et al.*, 2001).

Although genetic factors are believed to be an important component in the pathogenesis of Type 1 diabetes (She, 1996; Field, 2002), also non-genetic factors appear to be required for disease onset since concordance rates among identical twins are only 30–50% (Redondo *et al.*, 2001). Among the non-genetic factors, viral infections are one of the leading candidate agents involved in the pathogenesis of Type 1 diabetes and a variety of different human viruses have been reported to be associated with human Type 1 diabetes, including CoxsackieB virus, rubella virus, mumps virus and several Herpesviridae (Jun and Yoon, 2001). Cytomegalovirus (CMV), a double stranded DNA virus belonging to the β-herpes virus family, is one of the Herpesviridae thought to be involved in the pathogenesis of Type 1 diabetes. This widespread virus (seropositivity varies from 60 to 90%) (Numazaki, 1997) causes latent and persistent infection without clinical symptoms in the immunocompetent host. In the immunocompromised host, however, primary infections and reactivation of the virus may cause severe morbidity and mortality (Alford and Britt, 1990; Sinzger and Jahn, 1996). In the past decades, several reports have suggested the existence of a relationship between CMV infection and the development of Type 1 diabetes in humans (Ward *et al.*, 1979; Jenson *et al.*, 1980; Pak *et al.*, 1988). To date, however, a causal relationship between CMV infection and the development of human Type 1 diabetes is still to be shown. Studies on the relationship between CMV and Type I diabetes in

*Jan-Luuk Hillebrands and Nienke van der Werf contributed equally to this work.
†Corresponding author. Tel.: +31-50-3632530. Fax: +31-50-3632512. E-mail: j.rozing@med.rug.nl

ISSN 1740-2522 print/ISSN 1740-2530 online © 2003 Taylor & Francis Ltd
DOI: 10.1080/10446670310001626517

human subjects might be complicated by the fact that CMV infection usually occurs at childhood and that Type I diabetes is a disease of slow evolution in which the pathogenetic process might already start years before clinical diagnosis (Tarn *et al.*, 1987; Foulis *et al.*, 1997).

Recently, we were able to show a causal relationship between CMV-infection and the development of Type I diabetes using a well-established model of autoimmune diabetes in rats. Infection of Diabetes Prone (DP)-BB rats, which develop autoimmune diabetes spontaneously at the age of 10–14 weeks (Mordes *et al.*, 2001), with RatCytomegalovirus (RCMV, e.g. the rat equivalent of Human CMV (Bruggeman *et al.*, 1982; 1985b)), resulted in significant acceleration of the onset of diabetes (J.L. Hillebrands, submitted).

Here, we report on the possible underlying mechanism of RCMV-induced acceleration of onset of diabetes in DP-BB rats. We first analyzed whether RCMV can be detected in pancreatic tissue and isolated pancreatic islets after viral infection *in vivo*. Results indicate that although RCMV can be detected in the pancreas shortly after infection, pancreatic islets appear to be non-permissive for RCMV infection. This observation argues against direct cytolytic infection of pancreatic β-cells as the underlying mechanism of RCMV-accelerated onset of diabetes. Since rats are infected with RCMV by intraperitoneal (i.p.) injection, we analyzed the possible relation between peritoneal macrophages (pMΦ) and accelerated onset of diabetes after infection. RCMV infection resulted in a significant increase in the number of pMΦ shortly after infection. A fraction of these pMΦ was infected with RCMV. Depletion of pMΦ shortly after infection completely neutralized the accelerating effect of RCMV on the onset of diabetes.

Taken together, our data indicate that RCMV accelerates the onset of diabetes without direct cytolytic infection of pancreatic β-cells. pMΦ appear to play an important role in the process leading to accelerated onset of diabetes since depletion of this population results in neutralization of the accelerating effect of RCMV.

### RESULTS

### Diabetes Onset after RCMV Infection

To determine the effect of acute RCMV infection on the onset of diabetes in DP-BB rats, animals were infected with $2 \times 10^6$ pfu RCMV at the age of 35 days. As shown in Fig. 1, RCMV infection significantly accelerated the onset of diabetes (determined by the presence of hyperglycaemia; >16 mM) compared with both control groups ($p = 0.0164$, Kaplan–Meier log rank test). Severe insulitis, characteristic for the development of autoimmune diabetes, was detected in pancreatic tissue from all hyperglycaemic animals (data not shown). Since no differences were observed in the onset of diabetes in mock-infected and untreated animals, data from both groups were pooled.

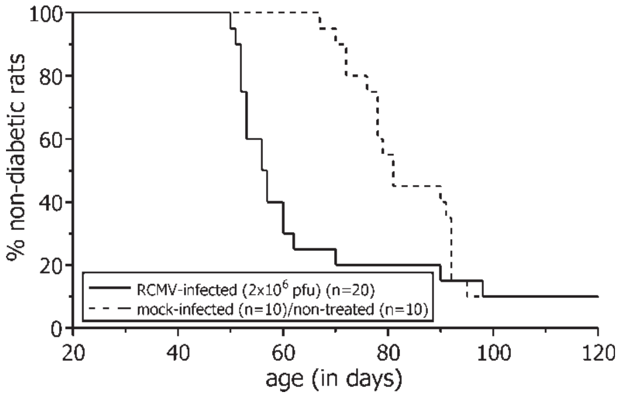

FIGURE 1 RCMV-infection ($2 \times 10^6$ pfu) of DP-BB rats at the age of 35 days significantly accelerates the onset of diabetes ($p = 0.0164$, Kaplan–Meier logrank test). Mock-infected and untreated animals showed similar onset of diabetes, and therefore data from both groups were pooled.

### Presence of RCMV in the Pancreas

To get more insight into the underlying mechanism by which RCMV accelerates the onset of diabetes, we first analyzed whether RCMV could be detected by specific immunohistochemistry in the islets in pancreatic tissue from animals which had become diabetic after infection. Since RCMV has a tropism for striated duct epithelial cells (Kloover *et al.*, 2000), staining on salivary gland tissue was used as a positive control. In the majority of the animals, however, only a few RCMV-infected cells (1–3) were detected in salivary glands, but occasionally high numbers (20–40) of infected cells were observed (Fig. 2A). In contrast to the salivary glands, RCMV-infected cells were never detected within the islets (Fig. 2B). As shown in Fig. 1, the first infected animals became already diabetic within 15 days after infection. It has been described that different peripheral organs, including the pancreas, become infected with RCMV, but primarily during the systemic phase of infection (±4 days–2 weeks p.i.) (Bruggeman *et al.*, 1985b). It is therefore not unlikely that we did not detect infected cells in pancreatic tissue because at that time the viral infection had already progressed to its latent phase, in which infected cells are predominantly detectable in the salivary glands. We therefore also analyzed pancreatic tissue for the presence of RCMV 7 days after infection. From half of the animals ($n = 3$) pancreatic islets were isolated, whereas from the remaining animals whole (non-dissociated) pancreatic tissue was used. Approximately 120 islets from each animal were used for DNA isolation. Since we expected to detect low numbers of infected cells we used a RCMV-specific nested PCR to detect viral DNA instead of less sensitive immunohistochemistry. Seven days p.i. RCMV was detected after first round PCR in non-dissociated pancreatic tissue from all RCMV-infected animals (Fig. 2C). As a negative control, the mock-infected animals were negative for viral DNA. In contrast

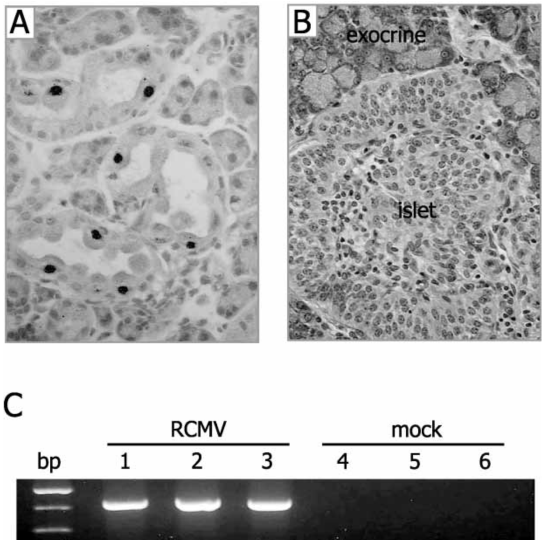

FIGURE 2 Detection of RCMV by immunohistochemistry and PCR analysis in salivary gland and pancreas of infected animals. (A) Photomicrograph showing RCMV-infected epithelial cells in the salivary gland of a diabetic DP-BB rat, 4 weeks after infection (original magnification: ×400). (B) Photomicrograph of pancreatic tissue of a diabetic DP-BB rat showing absence of RCMV-infected cells in islets 4 weeks after infection (original magnification: ×400). (C) RCMV-specific PCR analysis performed on non-dissociated pancreatic tissue from 3 RCMV-infected (1–3) and 3 mock-infected (4–6) rats 7 days after infection. All samples from RCMV-infected animals show the RCMV-specific PCR product, indicating that viral DNA is present in the pancreas 7 days after infection.

to non-dissociated pancreatic tissue, only 1 out of 3 animals showed a weak positive signal after second round PCR in the isolated pancreatic islet samples (data not shown). Since the nested PCR used is very sensitive (approximately 1 RCMV genome copy/800 cells) (Beisser *et al.*, 1998), these data suggest that although viral DNA is present in the pancreas 7 days p.i., pancreatic islets are relatively non-permissive for RCMV. The infected cells responsible for the positive signal in non-dissociated pancreatic tissue are not identified yet, but preliminary data suggest that infected infiltrating macrophages rather than infected exocrine tissue might be the source (data not shown).

### Numbers of Peritoneal Macrophages after RCMV Infection

Data obtained so far suggest that direct cytolytic infection of pancreatic islets is not the underlying mechanism of RCMV-accelerated onset of diabetes in DP-BB rats. Since infected macrophages were occasionally found in the pancreas early (< 1 week) after infection, it is likely that those cells contribute to the process of accelerated diabetes development. In our protocol, the standard route for RCMV-infection is by i.p. injection, and therefore we analyzed the role of peritoneal macrophages (pMΦ) in RCMV-accelerated onset of diabetes. First we analyzed the effect of viral infection on the absolute numbers of pMΦ that can be retrieved from the peritoneal cavity (PC) at different time points after infection. As shown in Fig. 3A, RCMV infection resulted in a significant increase in the number of pMΦ as rapid as 1 day after infection ($p = 0.036$ vs. mock-infection and $p = 0.0056$ vs. control [no treatment], 2-tailed Mann–Whitney test). At 3 and 6 days after infection, the numbers of pMΦ seemed to restore to normal (control) levels. Since immunohistochemical staining on cytospots showed that the cells isolated by peritoneal lavage almost exclusively consisted of $1C7^{+}$ macrophages at all time points studied (Fig. 3B), we conclude that the increase of cells 1 day after infection can be fully attributed to recruitment of $1C7^{+}$ phagocytes into the PC. RCMV-infected cells were consistently found at all three time-points (Fig. 3C), but the numbers were low (~0.05% of pMΦ). However, as will be discussed later, immunohistochemical detection of RCMV IE-antigens with Mab 8 may have resulted in a significant underestimation of the actual number of RCMV infected pMΦ.

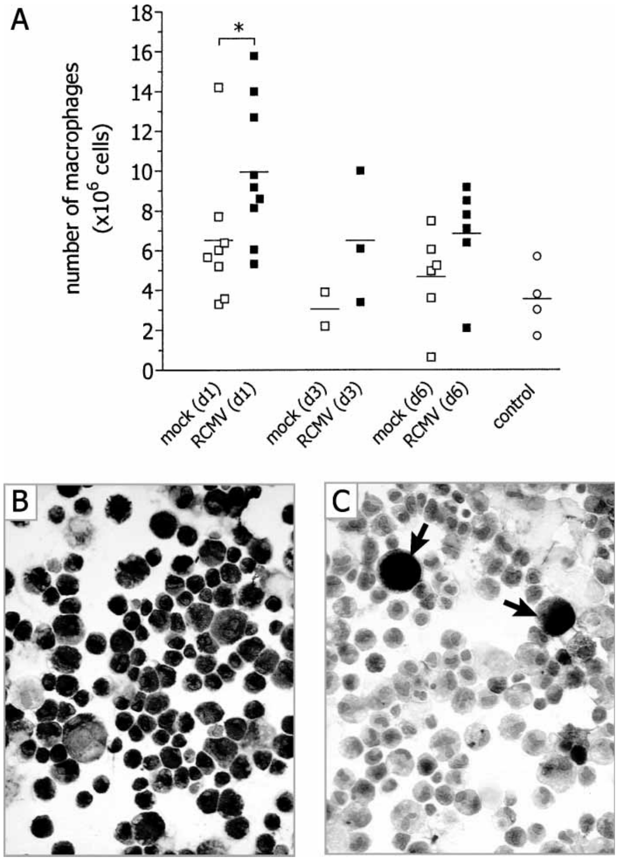

FIGURE 3 RCMV-infection results in increased numbers of pMΦ. Animals were i.p. infected with RCMV ($2 \times 10^6$ pfu), mock-infected or left untreated (control) and on days 1, 3 and 6 p.i. peritoneal lavage was performed and the number of cells were counted. (A) RCMV-infection resulted in a significant increase in the number of pMΦ 1 day after infection ($p = 0.036$ vs. mock infection and $p = 0.0056$ vs. control, 2-tailed Mann–Whitney test). At the later time-points the number of pMΦ restored back to normal levels. (B) Photomicrograph of cells isolated by peritoneal lavage. The cell population almost exclusively consisted of $1C7^{+}$ macrophages (original magnification: ×400). (C) Photomicrograph of cells isolated by peritoneal lavage containing $mAb8^{+}$ RCMV-infected cells (arrows) (original magnification: ×400).

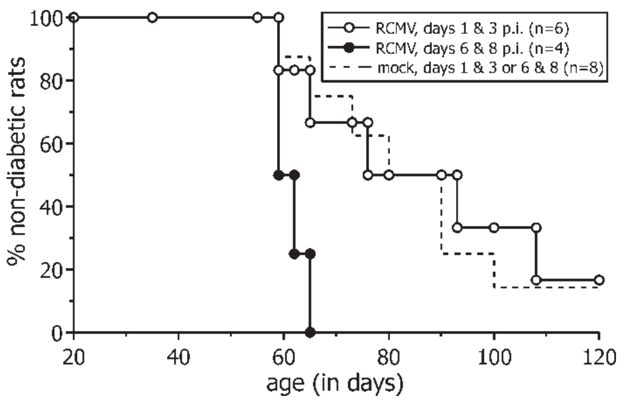

FIGURE 4 Depletion of pMΦ "early" after RCMV infection completely neutralizes the RCMV-induced acceleration of diabetes development. Animals were infected with RCMV ($2 \times 10^6$ pfu) or mock-infected. At "early" (days 1 and 3) and "late" (days 6–8) time points after infection, pMΦ were depleted by peritoneal lavage and the animals were followed for diabetes development. Peritoneal lavage "late" after infection did not neutralize the accelerating effect and animals developed diabetes in an accelerated fashion compared to the mock-infected and "early" lavage RCMV-infected rats ($p = 0.0056$ and 0.0223, respectively, Kaplan–Meier log rank test).

### Depletion of Peritoneal Macrophages after RCMV Infection

Since we showed that i.p. RCMV-infection results in increased numbers of peritoneal macrophages of which some are productively infected, we determined the effect of depleting macrophages "early" (days 1–3) and "late" (days 6–8) after infection on the RCMV-induced accelerated onset of diabetes. As is shown in Fig. 4, depletion of macrophages "early" after infection completely neutralized the RCMV-induced acceleration of diabetes development, and no significant differences were observed between RCMV-infected animals and mock-infected animals. (Data from mock-infected animals were pooled since no differences in the onset of diabetes were observed between "early" and "late" lavaged mock-infected animals). Peritoneal lavage on days 6 and 8 after infection, however, did not neutralize the accelerating effect of RCMV: animals undergoing "late" lavage still showed accelerated onset of diabetes compared to the mock-infected and "early" lavage RCMV-infected rats ($p = 0.0056$ and 0.0223, respectively, Kaplan–Meier log rank test).

### DISCUSSION

Natural perturbants like viral infections are believed to play a role in the pathogenesis of Type 1 diabetes. One of the candidate viruses is CMV, a herpes virus with a widespread distribution (Numazaki, 1997). Although some correlative studies on CMV and Type 1 diabetes have been reported, it turns out to be very difficult to show any causative relationship (Ward *et al.*, 1979; Banatvala *et al.*, 1985; Pak *et al.*, 1988; Foulis *et al.*, 1997). Recently, we described an animal model for Type 1 diabetes in which a direct association between CMV infection and onset of diabetes was observed: RCMV infection of DP-BB rats (which develop Type 1 diabetes spontaneously at the age of 10–14 weeks) resulted in accelerated onset of disease.

In this paper, we addressed the question what the mechanism could be behind this phenomenon of virus-induced accelerated onset of diabetes. One explanation might be that RCMV infection results in direct cytolytic destruction of pancreatic β-cells. Here we showed, however, that RCMV antigens could not be detected in the islets from infected diabetic rats, and we were only able to demonstrate presence of viral DNA in isolated pancreatic islets in 1 out of 3 samples. These data suggest that islets are non-permissive for RCMV infection and in line with this, preliminary data suggest that also cultured islets are non-permissive for RCMV infection *in vitro* (data not shown). Non-permissiveness of islets and β-cells for CMV infection has also been reported for human CMV (HCMV); only 1–2% of the β-cells in cultured fetal islets become infected after culturing in the presence of HCMV without direct destruction of the β-cells (Numazaki *et al.*, 1988, 1990). In addition, we showed that both co-isogenic Diabetes Resistant (DR)-BB rats as well as other rats strains tested do not develop diabetes after RCMV infection (J.L. Hillebrands, submitted). Taken together, these data indicate that direct cytolytic infection of pancreatic β-cells is not the mechanism by which RCMV accelerates the onset of diabetes.

Since RCMV-infected macrophages could be detected in pancreatic tissue (primarily located in the interlobular spaces) shortly after i.p. infection (<1 week), we wondered whether these cells are involved in the pathogenetic process of RCMV-induced acceleration of diabetes onset in DP-BB rats. As in our protocol, the standard route for administration of virus to achieve systemic RCMV infection is by i.p. injection (Bruggeman *et al.*, 1985b). Since the PC contains considerable numbers of leukocytes, predominantly consisting of resident macrophages (peritoneal macrophages, pMΦ) (Hendrix *et al.*, 1986), we studied the role of this macrophage subset in accelerated diabetes development. By analyzing the absolute numbers of pMΦ isolated by peritoneal lavage at different time points after infection, we showed that 1 day after infection the number of pMΦ present in the PC is significantly increased, most likely as a result of recruitment of mononuclear cells into the peritoneum. This increase in pMΦ numbers in response to RCMV infection was previously reported by Engels *et al.* who moreover showed that this influx of mononuclear cells was preceded by an influx of polymorphonuclear granulocytes (PMN), reaching maximum numbers already 2 h after infection (Engels *et al.*, 1989). At 1 day after infection we also observed increased numbers of PMN's, but the vast majority of isolated cells at this time point consisted of macrophages as determined by specific immunohistochemistry. At days 3 and 6 we observed a decrease in cell counts back to normal levels suggesting migration out of the PC. At all time points analyzed we could detect infected pMΦ using immunohistochemical detection of RCMV IE-antigens, although the percentages were low (~0.05%). This suggests that almost no reproductive infection occurs in pMΦ (Engels *et al.*, 1989). This does not exclude the possibility that many cells have taken up virus, however, without subsequent viral replication (i.e. the cells become latently infected). For HCMV it has been shown that monocytes can become latently infected without viral reproduction, and such cells are believed to play an important role in virus dissemination (Sinclair and Sissons, 1996; Michelson, 1997). Without having productive viral replication themselves, HCMV-infected monocytes have been shown to be able to transfer virus to more permissive cell types like fibroblasts (Waldman *et al.*, 1995). Moreover, HCMV can be reactivated (resulting in viral replication) if latently infected monocytes are activated and differentiate into macrophages (Ibanez *et al.*, 1991).

That pMΦ in infected DP-BB rats play an important role in the accelerated development of diabetes is shown by the fact that depletion of these cells early after infection results in complete neutralization of the accelerating effect of RCMV. It is unlikely that this is due to the removal of free, non-cellbound, virus from the PC since it has been demonstrated that RCMV is almost completely cleared from peritoneal lavage fluid within 8 h after i.p. infection (Bruggeman *et al.*, 1985a). Plaque-assays performed on lavage fluid isolated 1 day after infection confirmed the absence of non-cellbound virus (data not shown).

Based on the data described in this study we conclude that pMΦ play an important role in RCMV-induced acceleration of diabetes onset. The increased influx of mononuclear cells in the PC followed by restoration to normal levels suggest migration of (infected) cells throughout the body. Since preliminary data indicate that there is accumulation of infected macrophages in the pancreas, we suggest that these infected pMΦ are preferentially attracted to the pancreas. Since autoreactive T cells play a central role in the development of Type I diabetes, we propose that such RCMV infected pMΦ will further activate and recruite autoreactive T cells, thereby accelerating the diabetogenic process. This has been shown for a pancreatropic Coxsackievirus in NOD mice in which infection resulted in accelerated onset of diabetes if the β-cell autoreactive T cells had already accumulated in and around the islets at the time of viral infection (Serreze *et al.*, 2000). Besides this non-virus specific activation of autoreactive T cells, a virus-specific component might be involved. Recent data indicate that RCMV induces a very strong T cell proliferative response resembling the response pattern after polyclonal stimulation, leaving the possibility open that RCMV might directly activate autoreactive T cells resulting in accelerated onset of diabetes (van der Werf *et al.*, this issue).

Future studies will be directed at characterizing the factor(s) responsible for (preferential) migration of infected pMΦ to the pancreas. In addition, functional analyses on the interaction between infected macrophages and (autoreactive) T cells are needed to further elucidate the mechanism by which RCMV accelerates the onset of diabetes.

### MATERIALS AND METHODS

### Animals

Diabetes Prone (DP-BB/Wor) BB rats were bred at the central animal facility of the University of Groningen, The Netherlands. The breeding stock was originally provided by the University of Massachusetts, Worcester, MA, USA. The animals were raised under specific-pathogen-free (SPF) and viral-antibody-free (VAF) conditions and had access to water and food *ad libitum*. The experiments described in this study were approved by the university Ethical Board for Animal Studies.

### Infection with RCMV

For viral infection, 35 days old male and female DP-BB rats were injected intraperitoneally with 1 ml saline containing $2 \times 10^6$ pfu RCMV (Maastricht strain). The RCMV-stock consisted of salivary gland homogenates obtained from infected rats and prepared as described elsewhere (Bruggeman *et al.*, 1982). Control rats were mock-infected or left untreated. Mock-infection was established by i.p. injection of 1 ml saline containing an equal amount of salivary gland homogenate as for RCMV, but now prepared from tissue obtained from non-infected rats.

### Experimental Setup

To study the effect of RCMV-infection on the onset of diabetes, DP-BB rats were RCMV-infected ($n = 20$), mock-infected ($n = 10$) or left untreated ($n = 10$). Diabetic rats were killed at diagnosis and non-diabetic animals were killed at the end of the follow-up period (age 120 days). Pancreas and salivary glands were removed and processed for RCMV-specific immunohistochemistry. To analyze whether RCMV is able to infect pancreatic islets rats were RCMV ($n = 6$) or mock-infected ($n = 6$). Seven days after infection, pancreatic islets were isolated from three animals in each group, whereas from the other three animals complete (non-dissociated) pancreatic tissue was removed. Isolated islets and pancreatic tissue were subsequently analyzed for the presence of viral DNA using a RCMV-specific PCR. To study the role of pMΦ in RCMV-modulated onset of diabetes, the number of retrievable pMΦ from the PC by lavage was determined at several time-points (days 1,3 and 6) after RCMV- and mock-infection. Moreover, immunohistochemistry for macrophages and RCMV was performed on these isolated cells. To analyze whether removal of pMΦ influences the onset of diabetes after viral infection, pMΦ were removed early (days 1 and 3)

and late (days 6 and 8) after RCMV or mock infection. After the second lavage animals were followed for diabetes development.

### Diabetes

Animals were monitored twice a week for physical well-being and weight loss. If weight loss was detected, the animals were checked for hyperglycemia by glucose measurements in tail vein blood using a Reflolux®S glucose sensor (Boehringer Mannheim, Germany). If glucose levels exceeded 16 mM (288 mg/dl) animals were considered diabetic.

### Isolation of Pancreatic Islets

Pancreatic islets were isolated as described elsewhere (de Vos *et al.*, 1997). Briefly, after distending the pancreas with Krebs–Ringer–Hepes buffer the pancreas was excised, chopped and subsequently digested using incubation with collagenase (Sigma type XI, Sigma, USA). Islets were separated from exocrine tissue by centrifugation over a discontinuous dextran gradient and further purified by handpicking. The yield of pancreatic islets was approximately 225 islets per pancreas.

### Isolation of Peritoneal Macrophages (pMΦ)

Peritoneal macrophages were retrieved from the PC by lavage. Therefore, 10 ml sterile saline was injected into the PC. After spreading the saline throughout the PC by abdominal massage, the injected fluid was retrieved with a 16 gauge canula via a small incision (3 mm) after which the wound was closed with one suture. Generally, a volume of at least 8 ml was retrieved using this method. After centrifuging the cells (1200 rpm, 10 min) pellets were resuspended in PBS, counted and adjusted to a concentration of $5 \times 10^5$ cells per ml. Finally, cells were spun onto glass slides ($75 \times 10^3$ cells/spot) using a Cytospin (Shandon, United Kingdom), air dried, fixed in acetone for 10 min at room temperature and stored until immunohistochemical staining.

### Histologic Analysis

For detection of RCMV antigens, 7 μm Bouin-fixed paraffin embedded tissue sections (pancreas and salivary gland) were stained with RCMV monoclonal antibody (mAb 8) (Bruning *et al.*, 1987). Briefly, after deparaffination tissue sections were incubated with mAb8 for 60 min. Subsequently, sections were incubated with a second step horseradish peroxidase or alkaline-phosphatase conjugated rabbit anti-mouse antibody (DAKO A/S, Denmark) for 30 min. The chromogens diaminobenzidine (DAB) or Fast-Red were applied for 30 min, and subsequently sections were counterstained with Mayer's hematoxylin and cover slipped. For staining of pMΦ, cytospots were aceton-fixed and stained for macrophages and RCMV (mAb's 1C7 (van Goor *et al.*, 1988) and mAb8, respectively) as described above.

### Detection of RCMV DNA

To determine the presence of viral DNA in pancreatic tissue or isolated pancreatic islets, DNA was isolated using a DNA extraction kit (DNeasy Tissue Kit, Qiagen, Westburg B.V., The Netherlands). Subsequently, RCMV-specific (nested) PCR analysis was performed. The primer sequences and PCR conditions are described elsewhere (Beisser *et al.*, 1998). Second-round PCR was only performed on those samples that were negative after the first-round PCR. PCR products were analyzed by agarose gel electrophoresis and visualized by ethidium bromide staining.

### Statistical Analysis

To detect significant differences in diabetes onset, the Kaplan–Meier log rank test was performed. The 2-tailed Mann–Whitney test was performed to test for significant differences in numbers of peritoneal macrophages. Differences were considered significant when $p < 0.05$.

### *Acknowledgements*

This study was supported by a grant of the Dutch Diabetes Foundation (DFN99.028). The authors would like to thank Bart de Haan and Martijn de Groot (Surgical Research Laboratory, Department of Surgery, University of Groningen) for excellent technical assistance in isolating pancreatic islets.

### *References*

Alford, C.A. and Britt, J.W. (1990) "Cytomegalovirus", In: Fields, B.N. and Knipe, D.M., eds, *Virology*, 2nd Ed..

Banatvala, J.E., Schernthaner, G., Schober, E., De Silva, L.M., Bryant, J., Borkenstein, M., Brown, D., Menser, M.A. and Silink, M. (1985) "Coxsackie B, Mumps, Rubella, and Cytomegalovirus specific IgM responses in patients with juvenile-onset insulin-dependent diabetes mellitus in Britain, Austria, and Australia", *Lancet* **1**, 1409–1412.

Beisser, P.S., Vink, C., van Dam, J.G., Grauls, G., Vanherle, S.J.V. and Bruggeman, C.A. (1998) "The R33 G Protein-coupled receptor gene of rat cytomegalovirus plays an essential role in the pathogenesis of viral infection", *J. Virol.* **72**, 2352–2363.

Bruggeman, C.A., Meijer, H., Dormans, P.H., Debie, W.M., Grauls, G.E. and van Boven, C.P. (1982) "Isolation of a cytomegalovirus-like agent from wild rats", *Arch. Virol.* **73**, 231–241.

Bruggeman, C.A., Grauls, G. and van Boven, C.P.A. (1985a) "Susceptibility of peritoneal macrophages to rat cytomegalovirus infection", *FEMS Microbiol. Lett.* **27**, 263–266.

Bruggeman, C.A., Mijer, H., Bosman, F. and van Boven, C.P.A. (1985b) "Biology of rat cytomegalovirus infection", *Intervirology* **24**, 1–9.

Bruning, J.H., Debie, W.H., Dormans, P.H., Meijer, H. and Bruggeman, C.A. (1987) "The development and characterization of monoclonal antibodies against rat cytomegalovirus induced antigens", *Arch. Virol.* **94**, 55–70.

Engels, W., Grauls, G., Lemmens, P.J., Mullers, W.J. and Bruggeman, C.A. (1989) "Influence of a cytomegalovirus infection on functions and arachidonic acid metabolism of rat peritoneal macrophages", *J. Leukoc. Biol.* **45**, 466–473.

Field, L.L. (2002) "Genetic linkage and association studies of Type I diabetes: challenges and rewards", *Diabetologia* **45**, 21–35.

Foulis, A.K., McGill, M., Farquharson, M.A. and Hilton, D.A. (1997) "A search for evidence of viral infection in pancreases of newly diagnosed patients with IDDM", *Diabetologia* **40**, 53–61.

van Goor, H., Harms, G., Gerrits, P.O., Kroese, F.G., Poppema, S. and Grond, J. (1988) "Immunohistochemical antigen demonstration in plastic-embedded lymphoid tissue", *J. Histochem. Cytochem.* **36**, 115–120.

Greiner, D.L., Rossini, A.A. and Mordes, J.P. (2001) "Translating data from animal models into methods for preventing human autoimmune diabetes mellitus: caveat emptor and primum non nocere", *Clin. Immunol.* **100**, 134–143.

Hendrix, M.G.R., Bruggeman, C.A. and van Boven, C.P.A. (1986) "Alterations of the functional state of peritoneal macrophages during rat cytomegalovirus infection *in vivo*", *FEMS Microbiol. Lett.* **33**, 111–115.

Ibanez, C.E., Schrier, R., Ghazal, P., Wiley, C. and Nelson, J.A. (1991) "Human cytomegalovirus productively infects primary differentiated macrophages", *J. Virol.* **65**, 6581–6588.

Jenson, A.B., Rosenberg, H.S. and Notkins, A.L. (1980) "Pancreatic islet-cell damage in children with fatal viral infections", *Lancet* **2**, 354–358.

Jun, H.S. and Yoon, J.W. (2001) "The role of viruses in Type 1 diabetes: two distinct cellular and molecular pathogenic mechanisms of virus-induced diabetes in animals", *Diabetologia* **44**, 271–285.

Kloover, J.S., Hillebrands, J.L., de Wit, G., Grauls, G., Rozing, J., Bruggeman, C.A. and Nieuwenhuis, P. (2000) "Rat cytomegalovirus replication in the salivary glands is exclusively confined to striated duct cells", *Virchows Arch.* **437**, 413–421.

Michelson, S. (1997) "Interaction of human cytomegalovirus with monocytes/macrophages: a love-hate relationship", *Pathol. Biol. (Paris)* **45**, 146–158.

Mordes, J.P., Bortell, R., Groen, H., Guberski, D., Rossini, A.A. and Greiner, D.L. (2001) "Autoimmune diabetes mellitus in the BB rat", In: Sima, A.A.F. and Shafir, E., eds, *Animal Models of Diabetes* (Harwood Academic Publishers, Amsterdam, The Netherlands), pp 1–41.

Notkins, A.L. and Lernmark, Å. (2001) "Autoimmune type 1 diabetes: resolved and unresolved issues", *J. Clin. Investig.* **108**, 1247–1252.

Numazaki, K. (1997) "Human cytomegalovirus infection of breast milk", *FEMS Immunol. Med. Microbiol.* **18**, 91–98.

Numazaki, K., Goldman, H., Wong, I. and Wainberg, M.A. (1988) "Viral infection of human fetal islets of Langerhans. Replication of human cytomegalovirus in cultured human fetal pancreatic islets", *Am. J. Clin. Pathol.* **90**, 52–57.

Numazaki, K., Goldman, H., Seemayer, T.A., Wong, I. and Wainberg, A. (1990) "Infection of human cytomegalovirus and rubella virus of cultured human fetal islets of Langerhans", *In Vivo* **4**, 49–54.

Pak, C.Y., McArthur, R.G., Eun, H.M. and Yoon, J.W. (1988) "Association of cytomegalovirus infection with autoimmune type 1 diabetes", *Lancet* **2**, 1–4.

Redondo, M.J., Yu, L., Hawa, M., Mackenzie, T., Pyke, D.A., Eisenbarth, G.S. and Leslie, R.D. (2001) "Heterogeneity of type I diabetes: analysis of monozygotic twins in Great Britain and the United States", *Diabetologia* **44**, 354–362.

Serreze, D.V., Ottendorfer, E.W., Ellis, T.M., Gauntt, C.J. and Atkinson, M.A. (2000) "Acceleration of type 1 diabetes by a coxsackievirus infection requires a preexisting critical mass of autoreactive T-cells in pancreatic islets", *Diabetes* **49**, 708–711.

She, J.X. (1996) "Susceptibility to type I diabetes: HLA-DQ and DR revisited", *Immunol. Today* **17**, 323–329.

Sinclair, J. and Sissons, P. (1996) "Latent and persistent infections of monocytes and macrophages", *Intervirology* **39**, 293–301.

Sinzger, C. and Jahn, G. (1996) "Human cytomegalovirus cell tropism and pathogenesis", *Intervirology* **39**, 302–319.

Tarn, A.C., Smith, C.P., Spencer, K.M., Bottazzo, G.F. and Gale, E.A. (1987) "Type I (insulin dependent) diabetes: a disease of slow clinical onset?", *Br. Med. J. (Clin. Res. Ed)* **294**, 342–345.

de Vos, P., de Haan, B.J., Wolters, G.H., Strubbe, J.H. and van Schilfgaarde, R. (1997) "Improved biocompatibility but limited graft survival after purification of alginate for microencapsulation of pancreatic islets", *Diabetologia* **40**, 262–270.

Waldman, W.J., Knight, D.A., Huang, E.H. and Sedmak, D.D. (1995) "Bidirectional transmission of infectious cytomegalovirus between monocytes and vascular endothelial cells: an *in vitro* model", *J. Infect. Dis.* **171**, 263–272.

Ward, K.P., Galloway, W.H. and Auchterlonie, I.A. (1979) "Congenital cytomegalovirus infection and diabetes", *Lancet* **1**, 497.

